# Retraction Note to: Mutation pattern is an influential factor on functional mutation rates in cancer

**DOI:** 10.1186/s12935-017-0436-4

**Published:** 2017-07-05

**Authors:** Chuance Du, Xiaoyuan Wu, Jia Li

**Affiliations:** 1 Department of Urology, Ganzhou Hospital Affiliated to Nanchang University, Ganzhou, Jiangxi Province China; 2 Department of Rehabilitation, Ganzhou Hospital Affiliated to Nanchang University, Nan Chang, Jiangxi Province China; 3 Department of Thyroid and Breast, Shanghai Tenth People’s Hospital, Tongji University, Shanghai, 200072 China

## Retraction to: Cancer Cell Int (2016) 16:2 DOI 10.1186/s12935-016-0278-5

The authors are retracting this article [1] because they do not have ownership of the data they report. A formal investigation by the Institut de Biologie Intégrative de la Cellule (I2BC), Gif-Sur-Yvette, France, has found that the methods and analyses presented in Figures 1, 3 and 5 and all computer analyses were performed at the Institut de Biologie Intégrative de la Cellule (I2BC) and the data have been previously reported in [2]. All authors agree with this retraction.
